# Magnesium alloy transfected BMSCs-BMP-2 composite in repair of femoral head necrosis with assessment of visceral organs

**DOI:** 10.1186/s40064-016-3472-y

**Published:** 2016-10-22

**Authors:** Kaka A. A. Katiella, Zhang Yanru, Zhang Hui

**Affiliations:** 1 Institute of Clinical Anatomy, Southern Medical University-Guangzhou, Guangdong, 510515 People’s Republic of China; 2 Medical College of Zhengzhou University, School of International Education, Zhengzhou University, Zhengzhou, 450051 Henan People’s Republic of China; 3 Orthopedic Department, The First Affiliated Hospital of Zhengzhou University, Zhengzhou, 450052 Henan People’s Republic of China

**Keywords:** BMSCs, BMP2, Magnesium alloy, Femoral head, Rabbits

## Abstract

**Background:**

This study was designed to investigate the effect of BMSCs transfected BMP-2 composite with magnesium alloy rod in the repair of the femoral head necrosis in New Zealand white rabbits. Multifactorial but mostly traumatic, osteonecrosis of the femoral head account for 10 % of the 250,000 total hip arthroplasties done annually in the United States while its prevalence in most countries in not known. However, early intervention prior to collapse is critical to successful outcomes in joint preserving procedures.

**Methods:**

The pcDNA3.1 plasmid from cultured BMSCs was successfully transfected into BMSCs-BMP-2 by electroporation. Femoral head necrosis were established in 40 rabbits by liquid nitrogen freezing method. Animals were randomly divided into four groups (n = 10): Mg rod/BMSCs group, Mg rod group, BMSCs group, and blank control group. The composite of BMSCs-BMP-2 on Mg alloy rods were implanted respectively into the left femoral metaphysis of rabbits till the femoral head. Radiographic X-ray examination, histological hematoxilin and eosin (H&E) analysis and immunohistochemistry techniques were performed postoperatively; to observe and compare by the schedule; the newly formed bone and the degradation of the Mg rod at 6 and 12 weeks, sacrificing five animals at each time.

**Results:**

Twelfth week histological and immunohistochemical examinations showed complete magnesium alloy absorption in experimental and control group. H&E staining and immunohistochemistry showed obvious differences, Mg rod/BMSCs group having the best recovery than the other groups. BPM-2 level of gene expression of experimental group was also higher than those of controlled group.

**Conclusion:**

BMP-2 coated Mg alloy promotes the expression of bone growth factors at the implant in marrow of rabbits thus delaying femoral head necrosis and improving repair.

## Background

In recent years, with the rapid economic development and sudden change in people’s livelihood, the incidence of the osteonecrosis of the femoral head necrosis (ONFH) increased but yet remain unknown in most countries. Traumatic or none in its cause, ONFH results in reduced blood flow to the femoral head, marrow and bone necrosis and collapse of the femoral head, when blood flow is at least 20 % lower than that in the healthy control (Zhao et al. 2007; Sunagawa et al. 2000). Clinically, the treatment of the ONFH in the early stage is recommended. However, relief on the choice of metal materials, compared with other metal implants, often as internal fixation material is more on magnesium a lightweight metal (Nassif and Ghayad 2013; Terada et al. 2009; Pettersen et al. 2002) and magnesium alloy. Current metallic biomaterials are essentially neutral in vivo, remaining as permanent fixtures, and metals are more suitable for load-bearing applications, and magnesium is close in physical properties of bone cortex (Nagels et al. 2003) and is essential to human metabolism and naturally found in bone tissue (Hartwig 2001). To predict any possible release of toxic metallic ions and/or particles through corrosion or wear processes (Lhotka et al. 2003; Jacobs et al. 2003) and avoid tissue loss (Wang et al. 2002), lung, liver, and kidney tissue samples were processed and analyzed as well.

## Methods

### Animals

Forty-two New Zealand white rabbits of both genders (weight 3–3.5 kg, age 4 months) were obtained from the Experimental Animal Center of Zhengzhou University. Animals were allowed to range freely single in their labeled cage and feed with a standard diet ad libitum. Surgeries were all performed under sterile conditions. All experiments were approved by the University branch of Local Institutional Animal Care and Use Committee and complied with the Guide for the Care and Use of Laboratory Animals (1996). Femoral heads necrosis was established in forty rabbits following liquid nitrogen freezing method by Yang et al. (2001). Animals were randomly divided into four groups (n = 10): magnesium rod/BMSCs group, magnesium rod group, BMSCs group, and blank or control group. Two separate rabbits were used for BMSCs culture.

### Alloy and plasmid

**Fig. 1:**
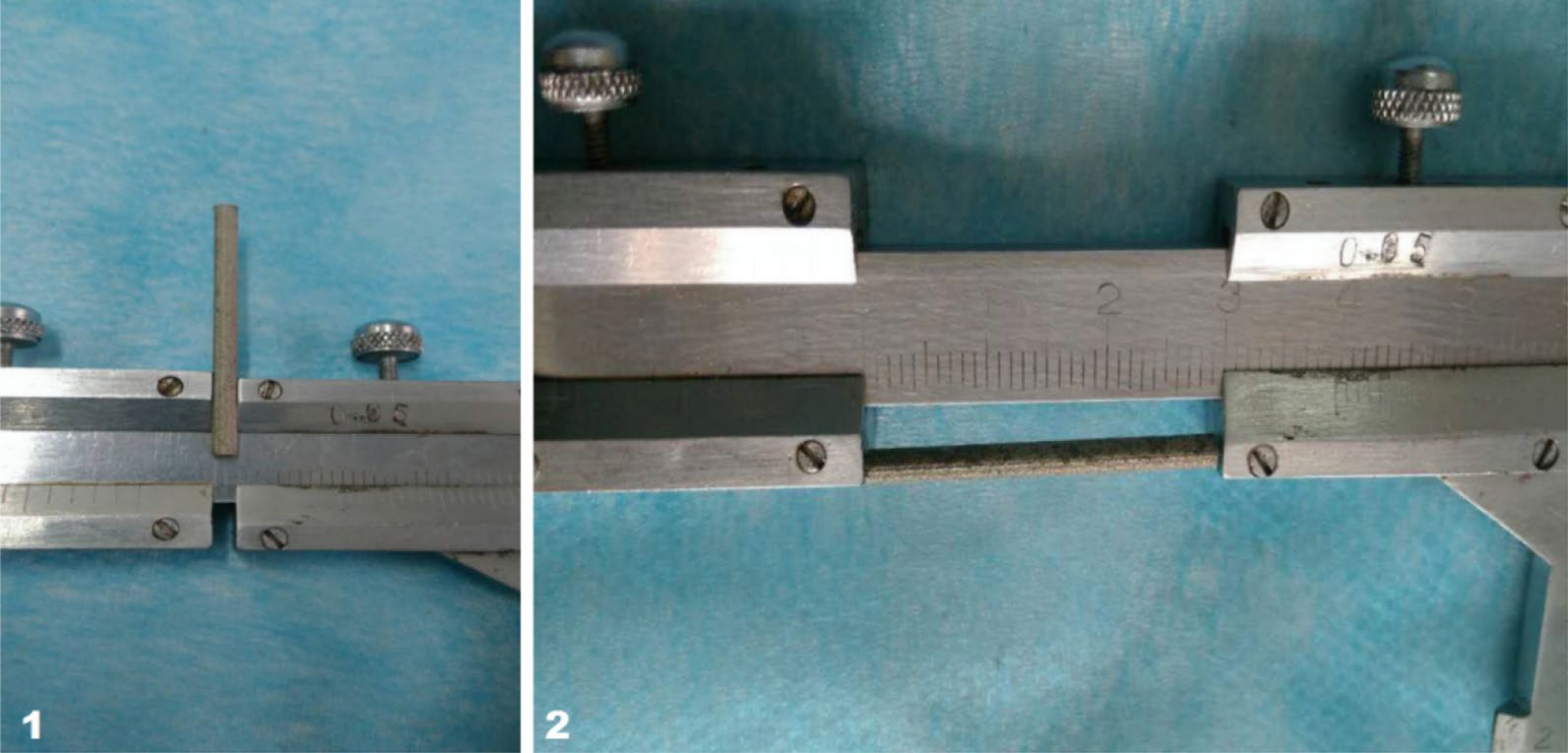
Magnesium alloy rod. **1** 2 mm diameter, **2** 30 mm length

The density of light metal magnesium was 1.749 cm^−1^, and the compressive yield strength (65.100 MPa). The magnesium alloy rod had 2 mm diameter, 30 mm length and composed of Mg-4/Zn-0.5/Ca-0.5/Al-1Nd purchased from Shanghai Research Institute of Materials (SRIM, Shanghai, P. R. China). The PcDNA3.1-BMP-2 plasmid (safely kept at the Clinical Skill Training Center of the Medical College, Zhengzhou University) was constructed by Vector Gene Technology Company (Beijing, China). Verified by DNA sequencing; the encoding BMP-2 was generated from plasmid pcDNA3.1-BMP-2 obtained from School of Basic Medical Sciences, Zhengzhou University (Fig. 1).

#### Femoral head necrosis

The animal models for the femoral head necrosis with liquid nitrogen freezing method was established after anesthesia with pentobarbital at a dose of 40 mg/kg. Under aseptic conditions, a 2 cm incision was made along the lateral aspect of the hip. Respectively, the superficial fascia, the gluteal muscles and the hip joint capsule was retracted and femoral head exposed. The ligaments were protected with carefully positioned retractors to prevent surrounding soft tissue mostly vessels damage. Medical gauze was used to protect the surrounding tissues from any liquid nitrogen spill. Using a sterile rubber funnel on the femoral head, a medical cotton swabs dipped in liquid nitrogen was applied freezing hip weight-bearing area for 3 min, and then thawing for 30 s. The procedure was repeated three times. Afterwards, tissue layers were sutured. Magnesium rods transfected with BMSCs were implanted into BMSCs group and Magnesium rod group. Postoperatively, animals were injected intramuscularly and eight hourly, Gentamicin at a dosage of 2 mg/kg for three consecutive days. Each animal was placed back in their single cage and allowed for safe recovery. Proper care for wound dressing and hygiene was maintained. Respectively at sixth week and twelfth week, five rabbits in each group were sacrificed under anesthesia (2 % sodium pentobarbital).

### Statistical analysis

Data are reported as mean ± SEM. Statistical analysis was performed using Student’s *t* test for unpaired data. *P* < 0.05 was considered significant.

## Results

### Clinical observations

All animals recovered from anesthesia and were allowed free water and food. Through the 12 weeks study duration, healing progressed monotonously in all animals; no infection noticed, no any postoperative complication was recorded during the observation periods. Hip joint activity was restored.

### Inter-group comparison

A week later, wound healing was good for all animals except for the blank control group which had a very poor recovery. However, each animal wound inflammation faded out after 6 weeks. Yet for the blank control group, improvement was limited. Magnesium rod group showed slight claudication. Magnesium rods/BMSCs group was best in recovery with perfect running ability. At twelfth week, the control group was still in lameness with poor mobility; and among the magnesium rod group only one animal was registered with slight claudication. In the BMSCs group three animals were having passable activity and slight limping. In Magnesium rod/BMSCs group all animals had regained normal activities. According to the behavioral observation of experimental animals, the effect of the Mg/BMSCs group had the best improvement.

### Macroscopic observations

Six weeks after alloy insertion, the graft appeared unremarkable. No border between graft and host bone. The metaphyseal cancellous bone appeared stable around the graft area. The cortical diaphyseal bone diameter was apparently increased but the shape was normal cylindrical and smooth. Twelfth weeks after surgery, the alloy was completely absorbed. Only spongy bone was observable. The proximal diaphyseal cortex appeared obviously increased in diameter.

### Radiological investigation

**Fig. 2:**
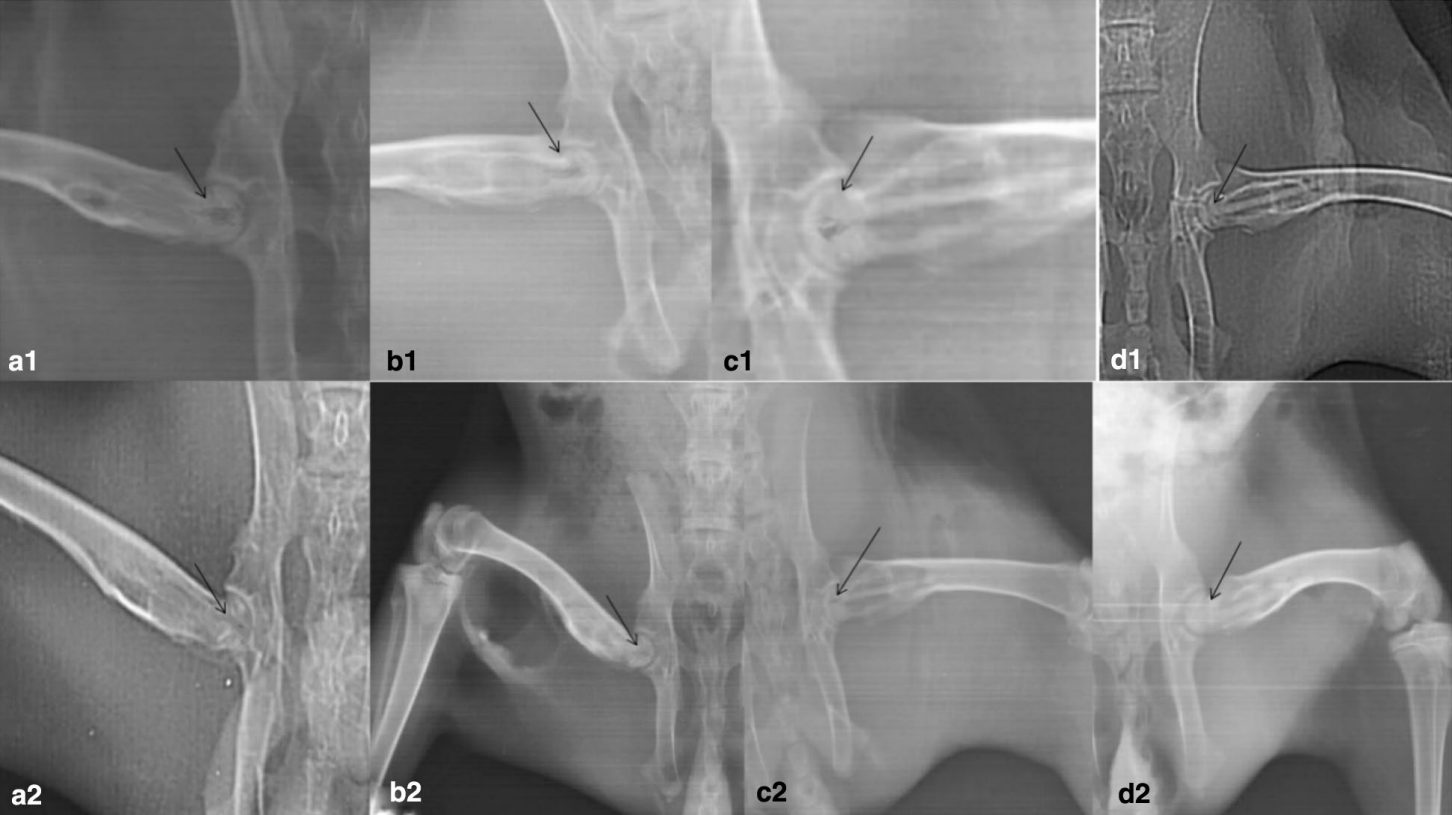
Radiography of all groups: In the untreated group, femoral head has collapsed with an abnormal contour and increased density (see *arrow* in **a1**, **a2**). BMSCs group (**a2**) low density shadow, centrally observable necrosis of femoral head. Magnesium rod group (**b2**) no necrosis, looming magnesium alloy, less obvious low density area as show the *arrows*. In **c1**, **c2**, **d1**, **d2** the femoral head has not collapsed. The density and the shape are nearly normal. The magnesium rod/BMSCs group (**c2**) shows a better bone mineral density (see *arrow* in **d2** vs. **c2**). Blank control group (**a2**) necrotic collapsed femoral head. Post operation first day: **a1**, **b1**, **c1**, **d1**. Pre-operation at twelfth week: **a2**, **b2**, **c2**, **d2**

The empty control and the alloy implant positions were checked subsequently under the postoperative anesthetic effect in Radiology Department, Third Affiliated Hospital of Zhengzhou University (Fig. 2). The implant was inserted after drilling the femoral cortex with a Tungsten drill 1/8″ at a point about 30 mm from the greater trochanter and approximately 13 mm from the lesser trochanter.

### Histological observations

#### Sixth week

In the graft, vascular proliferation was observed including some osteoclastic activity. In the alloy area were seen few lamellar necrotic tissue, fine vascular proliferation, and enlarged Haversian canals (Fig. 12). As implant absorption takes place, osteoclast activity was undisrupted.

#### Twelfth weeks

**Fig. 3:**
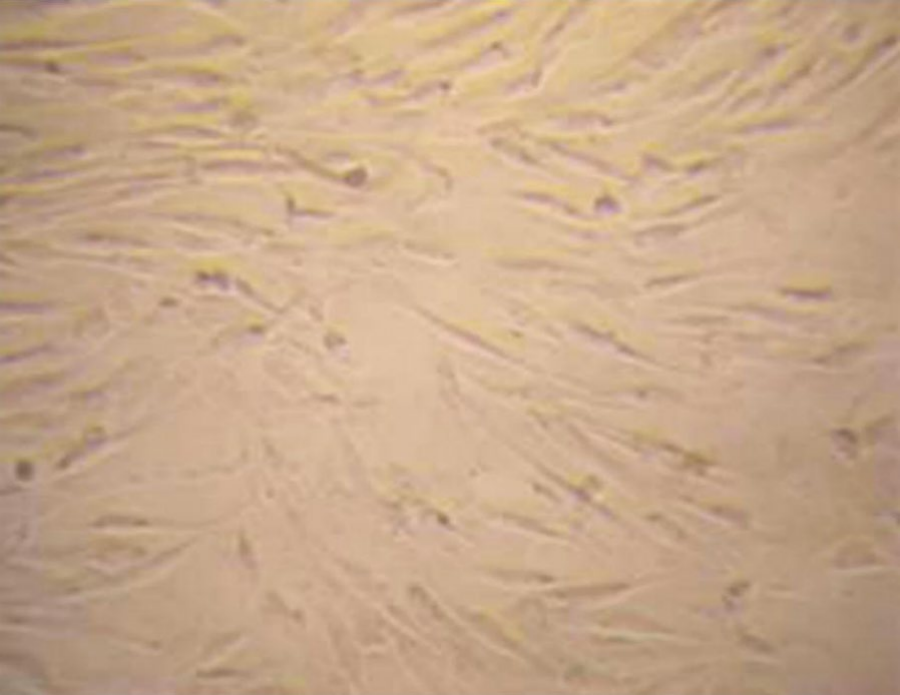
Identification of osteogenic cells derived from the BMSCs. Phase-contrast microscopic images of cultured rabbit BMSCs (×100)

**Fig. 4:**
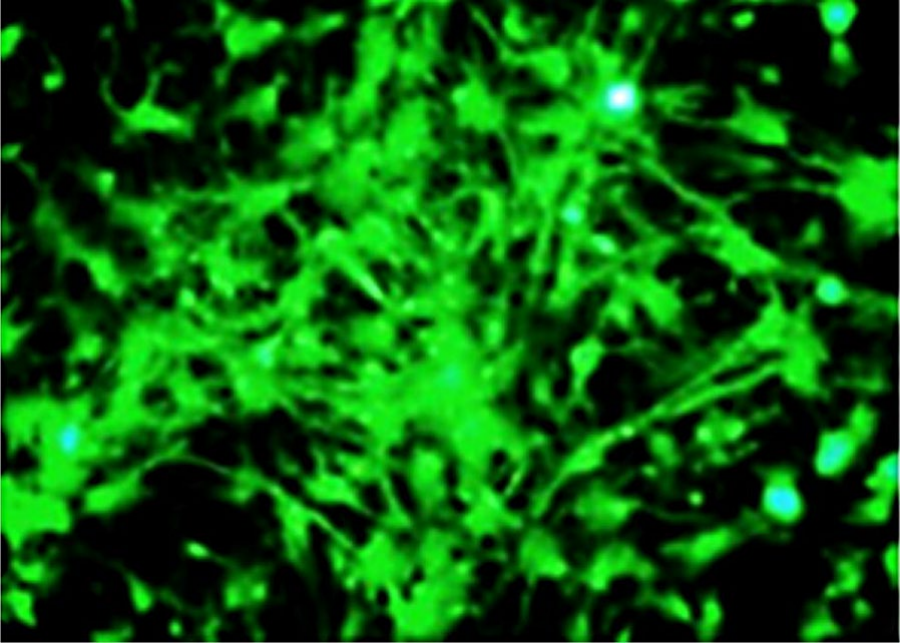
During osteoblastic differentiation, successfully transfected BMSCs pcDNA3.1-BMP-2 shows osteogenic cells with elevated type I collagen expression (×100)

**Fig. 5:**
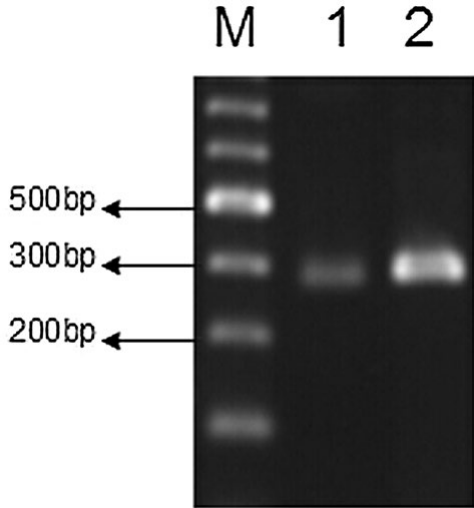
RT-PCR detection of mRNA BMP-2 expression (*1* pcDNA3.1-BMP-2 was not transfected; *2* BMSCs of the recombinant vector was transfected into BMSCs)

**Fig. 6:**
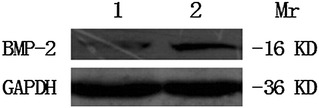
BMP-2 expression by Western blot method

**Fig. 7:**
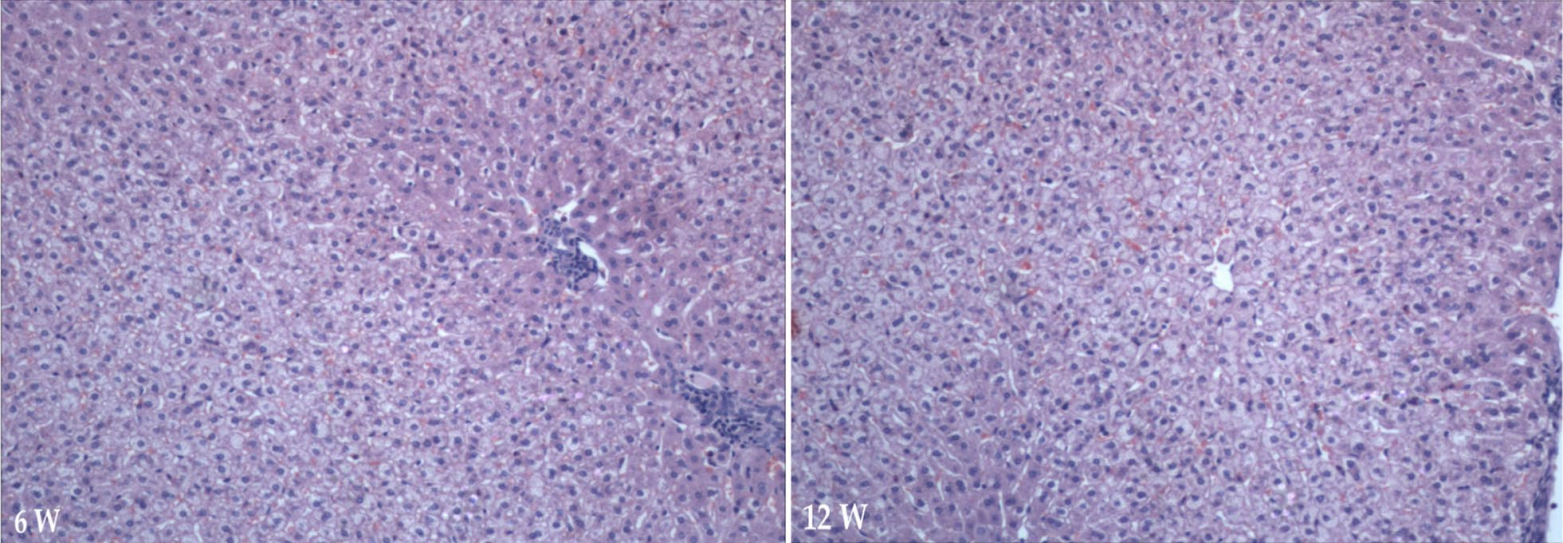
H&E Identification of liver tissue. Liver cells with mild steatosis, phase-contrast microscopic ×100 (*6w* week six, *12w* week 12)

**Fig. 8:**
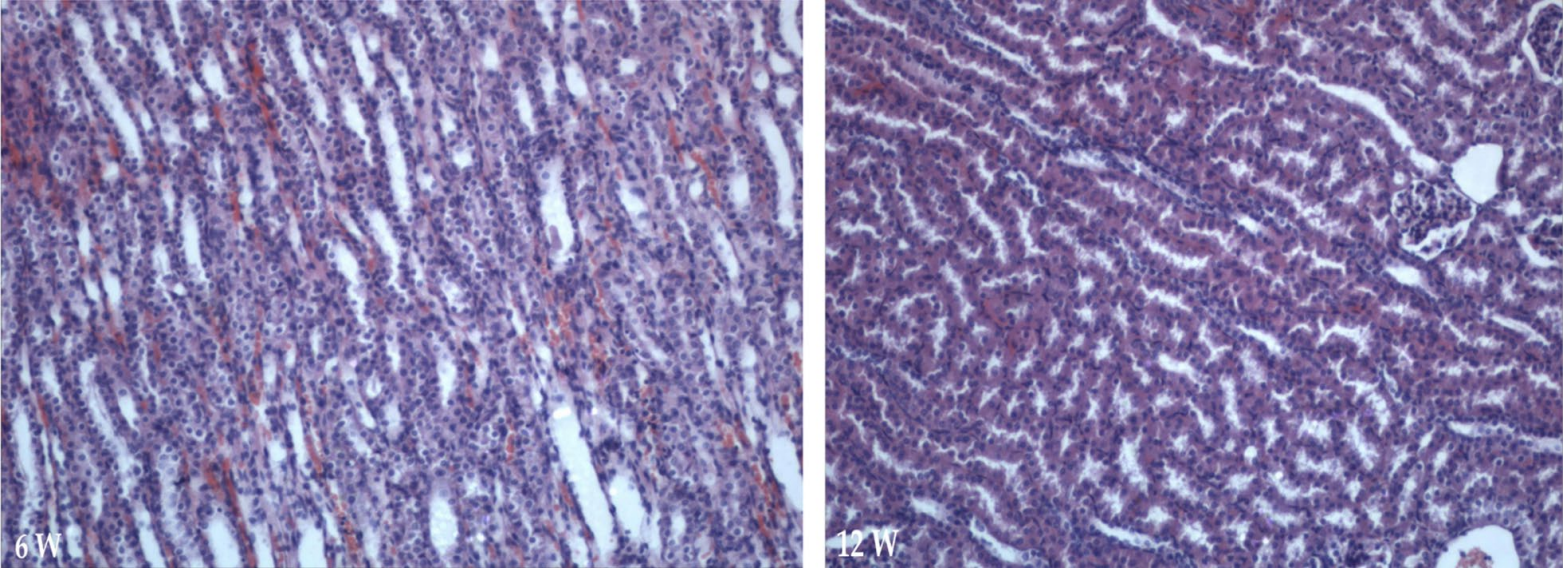
H&E Identification of kidney tissue, phase-contrast microscopic ×100 (*6w* week six, *12w* week 12)

**Fig. 9:**
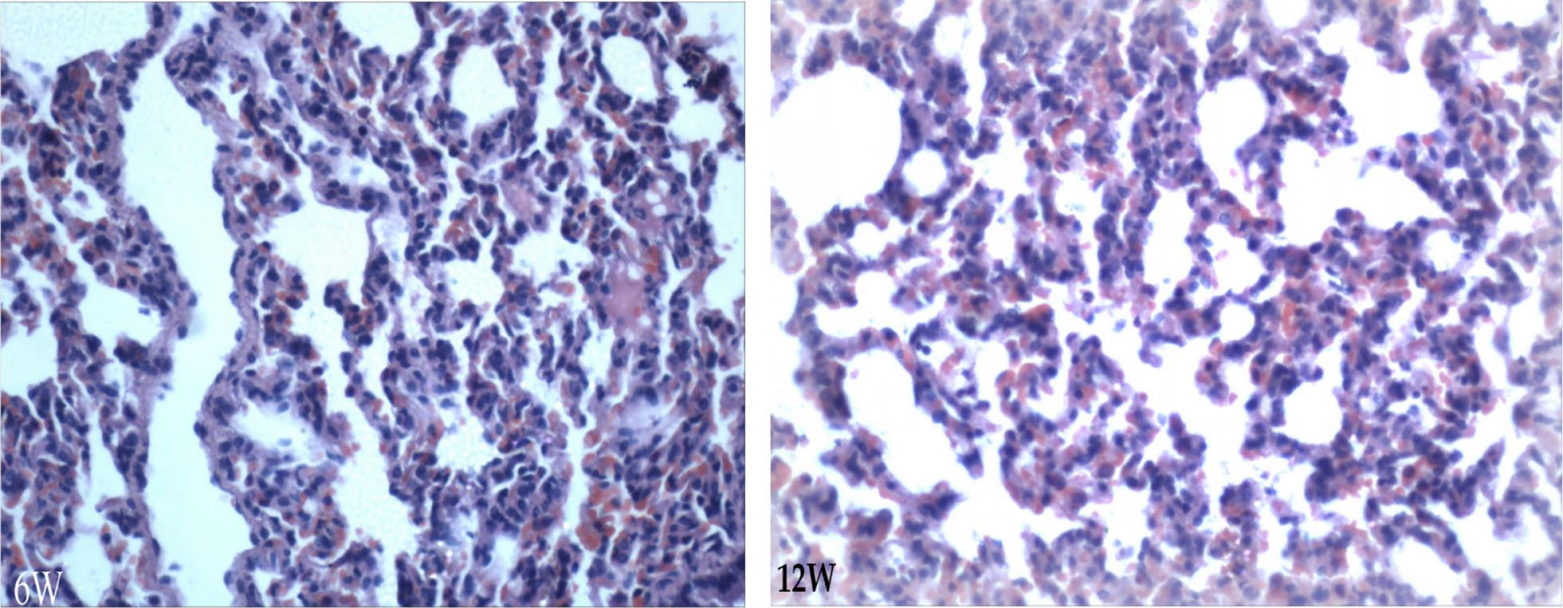
H&E Identification of lung tissue, phase-contrast microscopic ×100 (*6w* week six, *12w* week 12)

**Fig. 10:**
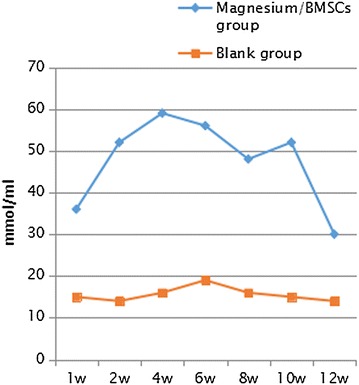
Magnesium ion concentrations in blood samples at weeks 1, 2, 4, 6, 8, 10 and 12 (*w* week)

**Fig. 11:**
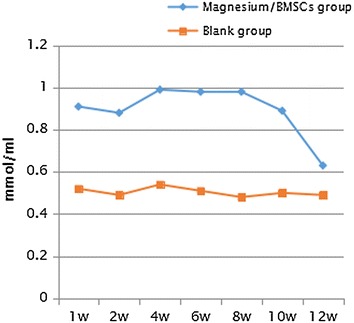
Magnesium ion concentrations in urine samples at weeks 1, 2, 4, 6, 8, 10 and 12 (*w* week)

**Fig. 12:**
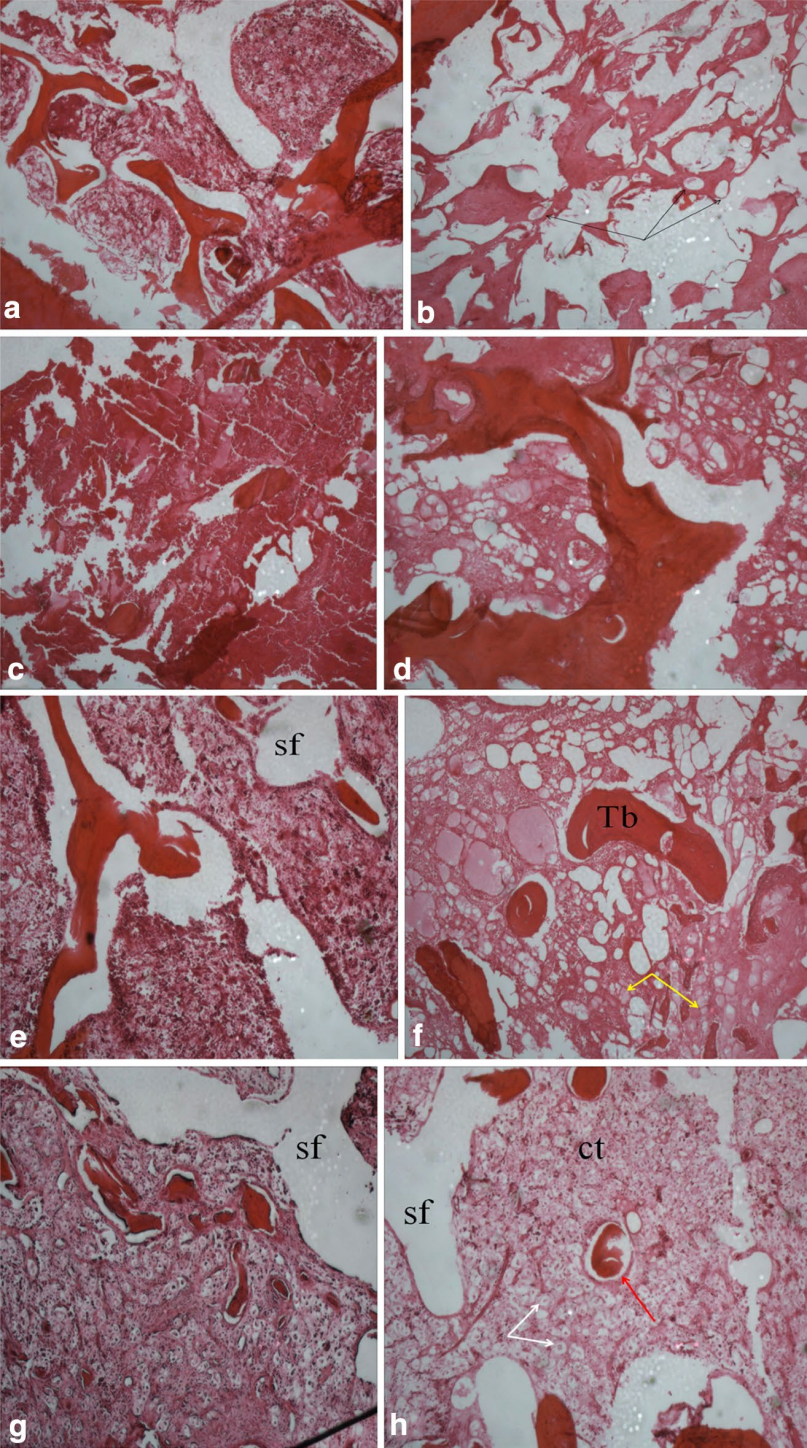
H&E staining observations-phase-contrast microscopic (×40). *Sixth week* Reduced hematopoietic cells in both BMSCs group (**a**) and magnesium rod group (**c**). Magnesium rod/BMSCs group (**e**) having and; blank group (**g**) with much necrotic cells, many lymphocytes [*yellow arrow*] and broken trabecular. *Twelfth week* BMSCs group (**b** with group less giant cells [*black arrow*]) and magnesium rod group (**d**) both showing irregular bone trabecular arrangement, few new osteoids, hematopoietic hyperplasia. Magnesium rod group/BMSCs (**f** with more endocytic cells: *white arrow*) has more arranged trabecular, no obvious implant area borders, very minimal difference with normal cancelous bone. Blank group (**h**) with visible necrotic cells

Osteoclasts activity was effective with the presence of the canalicculi. Necrostic cells were significantly decreased. Endosteal proliferation was obvious (Fig. 12). Osteoblasts functions were also observed with few new bone formations as seen in the improved femoral head density (Figs. 3, 4, 5, 6, 7, 8, 9, 10, 11, 12). Metaphyseal cancellous bone was normalized, alloy completely disappeared. The bone matrix deposition around the. Though there was obvious slight increase in proximal diaphysis, no histological difference was observed in the alloy area. At twelfth week, although we did not assess the density and level of resilience of the host bone, there was an excellent healing process in the medullary cavity.

## Discussion

Osteonecrosis is a disease of unknown pathogenesis that usually progresses to hip joint destruction necessitating total hip arthroplasty. The pathology involves ischemic events followed by death of bone and marrow elements (Mont et al. 1998). Because the results of hip arthroplasty in patients with osteonecrosis are relatively poor, much focus has been on modalities aimed at femoral head preservation (Xiaobing et al. 2015). Thus, to succeed in establishing that, the liquid nitrogen technique, easier and convenient, was used in this study because it achieves a high success rate with reliable osteonecrosis and a short pathological process and the basic properties of the bone are maintained (Berglund et al. 2016; Huang et al. 2013). In this study, the ONFH was properly established with the liquid nitrogen technique used, leading to a collapse of the femoral head in the control group and restoration of the femoral with perfect osteoids formation in the treatment group.

The BMSCs derived from rabbits contain a rich source of osteoprogenitor cells, and therefore are an abundant source of seeding cells for tissue engineering. Osteogenic growth factor here bone morphogenetic protein-2 (BMP-2) has been demonstrated to facilitate the bone regeneration in a critical size defect model while the in vivo osteogenic differentiation of BMSCs could lead to bone formation under certain conditions (Xia et al. 2012; Arrabal et al. 2013; Skogh et al. 2013). In the environment of high chloride ion and animal body (ph 7.4–7.6), magnesium and magnesium based materials have good biodegradability. In our study, at week 16 after surgery, the magnesium allow was completely absorbed at the implant site. It was reported that, high concentrations of magnesium are toxic, and serum magnesium levels above 1.05 mmol/L may lead to muscle paralysis, low blood pressure, respiratory distress. Serum magnesium concentration reaches 6–7 mmo1/L, which can cause cardiac arrest. Magnesium produces a large amount of hydrogen, which can not be absorbed in the body, leading to severe emphysema when the corrosion rate of magnesium is relatively high. However, we found safe the use of magnesium alloy in the assessed tissues as indicated in the “Results” section. Nonetheless, magnesium deficiency is associated with increased contractility of smooth muscle cells and could lead to bronchial smooth muscle contraction or lack of bronchial muscle relaxation (de Valk et al. 1993). Contrary to the latter, in this study we found that metaphyseal cancellous bone was normalized and the magnesium alloy accurately measured was nontoxic and was successfully absorbed. The assessment of visceral organs were safe and corrosion resistant. This is further reported in the findings of Vormann (2003). As the degradable alloys (mainly magnesium and iron based alloys) are expected to degrade inside human body, the main compositions of the alloys should be metallic elements that can be metabolized, and demonstrates appropriate degradation rates in the human body (Zheng et al. 2014). Toxicity and absorption ability depend on the composition of the alloy. Compared to polymer-based materials, biodegradable metals have higher stiffness and strength, and are more suitable for load bearing conditions (Staigera et al. 2006) which the restored ONFH in the treatment group is a proof for the selection of magnesium alloy. Magnesium is the fourth most abundant cation in the body and the second most common intracellular cation, and it intervenes in calcium transport mechanisms, intracellular phosphorylation reactions. It constitutes an important determinant of the contraction and relaxation state of bronchial smooth muscle (Dominguez et al. 1998) and about 60 % of the magnesium is present in bone and has a 4.5 mg/kg/day recommended daily allowance in adults while the literature recorded previously 6–10 mg/kg/day (Seo and Park 2008).

So the use of alloy fits better based on its characteristics because, the inclusion of porosity results in a material with reduced yield strength and modulus, corresponding with the lower range of mechanical properties of natural bone (Wen et al. 2004). The cortical diaphyseal bone diameter was apparently increased but the shape was normal cylindrical and smooth at the sixth week; the twelfth week showed only spongy bone and the increased proximal diaphyseal diameter was maintained. Although, porous metals were found suitable to be adjustable on demand (Wang et al. 2016); it has also been proved that, the degradable magnesium alloy bone screws were found clinically equivalent to the conventional Ti screws; and no foreign body reaction, osteolysis, or systemic inflammatory reaction were observed for the Mg alloy screws (Windhagen et al. 2013). Therefore, alloying is an essential step to improve mechanical properties and corrosion resistance of magnesium and safe in vivo (Zhuang et al. 2016).

During bone growth and repair, bone formation is initiated and supported by blood vessels. Osteoblasts differentiate and proliferate around the vessel, arrange along the vascular endothelium and then excrete osteoids in a direction away from the vessel. When the osteoblasts mature and develop into osteocytes, the newly formed bones deposit around the vessel (Huang et al. 2013). Bone is a complex tissue that continually undergoes dynamic biological remodelling, i.e. the coupled process whereby osteoclasts resorb mature bone tissue followed by osteoblasts that generate new bone to maintain healthy homeostasis of bone (Wang et al. 2016), yet our next study will assess the observed increase in proximal diameter of the femoral shaft. We believe that magnesium alloy and BMScs-BMP-2 are promising easy materials which may require a weekly assessment in other experimental animals but for a long time study period to clear all bias on the toxicity of magnesium and its ally with BMP-2 is promising for repair of the ONFH.

## Conclusion

It will be safer and more excellent to evaluate the magnesium and it alloys in vivo than in vitro due to the toxicity effect reported by few authors. This study showed that magnesium alloy is stable and perfectly absorbed with its BMSCs-BMP-2 composite’s potentials in promoting new bone formation for repairing the femoral head necrosis in rabbits. No any obvious complication nor damage was found over the investigated viscerals: liver, lung, kidney. Based upon the result of this study, due to the slight increase in femoral proximal cortical diameter, further investigation utilizing larger sample size and longer evaluation intervals in rabbits and other animals is wanted for proper clinical applications. In addition possible correlation with histomorphometric and quantitative measurements of Mg in blood plus its concentration in the various visceral organs should be investigated.
